# Induced pluripotent stem cells from sporadic pulmonary veno-occlusive disease exhibit altered endothelial commitment and stress-related transcriptional programs

**DOI:** 10.3389/fcell.2026.1811995

**Published:** 2026-08-03

**Authors:** Ling Lian, Noah Daniels, Nicholas Wanner, Suzy Comhair, Gunnar H. D. Poplawski, Kewal Asosingh, Rodrigo Lopez-Gonzalez

**Affiliations:** 1 Department of Neurosciences, Cleveland Clinic Research, Cleveland, OH, United States; 2 Department of Inflammation and Immunity, Cleveland Clinic Research, Cleveland, OH, United States; 3 Center for Immunotherapy and Precision Immuno-Oncology, Cleveland Clinic Research, Cleveland, OH, United States

**Keywords:** endothelial differentiation, induced pluripotent stem cells (iPSCs), oxidative stress, pulmonary veno-occlusive disease (PVOD), transcriptomics

## Abstract

**Introduction:**

Pulmonary veno‐occlusive disease (PVOD) is a rare form of pulmonary hypertension characterized by progressive vascular remodeling, with limited understanding of its cellular and molecular mechanisms, particularly in sporadic cases lacking identifiable genetic mutations. We investigated whether patient‐derived induced pluripotent stem cells (iPSCs) recapitulate disease‐associated cellular phenotypes and transcriptional alterations in sporadic PVOD.

**Methods:**

Integration‐free iPSCs were generated from a patient with sporadic PVOD and differentiated toward endothelial and hematopoietic lineages. Endothelial differentiation capacity was assessed by flow cytometry, and transcriptomic profiling was performed using RNA sequencing followed by differential gene expression and gene set enrichment analyses.

**Results:**

PVOD‐derived iPSCs demonstrated preserved hemangioblast generation but reduced endothelial commitment compared with control cells. Transcriptomic profiling revealed clear segregation between PVOD‐derived and control samples by principal component analysis, indicating distinct global gene expression patterns. Differential expression analysis identified genes associated with cellular stress and metabolic regulation, while gene set enrichment analysis demonstrated enrichment of reactive oxygen species and transforming growth factor‐β (TGF‐β) signaling pathways, consistent with altered stress‐related transcriptional programs.

**Discussion:**

These findings indicate that iPSCs derived from a patient with sporadic PVOD exhibit altered endothelial differentiation and distinct stress‐related transcriptional signatures. This patient‐specific iPSC model provides a platform for investigating disease‐associated cellular alterations in sporadic PVOD.

## Introduction

Pulmonary veno-occlusive disease (PVOD) is a rare and life-threatening subtype of pulmonary hypertension characterized by progressive obstruction and remodeling of the pulmonary venous vasculature, leading to elevated pulmonary arterial pressures, severe hypoxemia, and right heart failure (Montani et al., 2016; Boucly et al., 2024). The clinical course of PVOD is often rapidly progressive, with lung transplantation remaining the only definitive therapeutic option (Humbert et al., 2022). Unlike other forms of pulmonary arterial hypertension, PVOD patients frequently respond poorly to vasodilator therapies, which may precipitate pulmonary edema, underscoring fundamental differences in disease biology and vascular involvement (Humbert, 2013).

Histopathologically, PVOD is marked by endothelial dysfunction, venous intimal thickening, extracellular matrix accumulation, and aberrant vascular remodeling (Deshwal et al., 2025). Both sporadic and heritable forms of the disease have been described, with biallelic mutations in *EIF2AK4* representing the most well-established genetic cause in familial cases (Eyries et al., 2014; Montani et al., 2017). However, a substantial proportion of patients present with sporadic disease in the absence of known pathogenic variants, highlighting the need for experimental systems capable of capturing non-genetic and stress-related contributors to disease pathogenesis.

Human induced pluripotent stem cells (iPSCs) provide a versatile platform for modeling disease-associated cellular phenotypes, particularly in rare disorders where access to primary tissue is limited (Grandy et al., 2019). Patient-derived iPSCs retain the genetic and epigenetic background of the donor and can be differentiated into relevant progenitor and lineage-committed cell types, enabling the investigation of early cellular abnormalities that may precede overt tissue pathology. While iPSC-based models have yielded important insights into pulmonary arterial hypertension, relatively few studies have focused on PVOD, and existing reports have largely emphasized endothelial proliferation phenotypes associated with *EIF2AK4* mutations (Gu et al., 2017; Ma et al., 2022).

In addition to lineage-specific defects, emerging evidence suggests that cellular stress responses and metabolic dysregulation contribute to pulmonary vascular disease, including altered redox homeostasis and mitochondrial function (Rabinovitch et al., 2014; Gu et al., 2017; Napoli et al., 2019). However, the contribution of these processes to sporadic PVOD remains incompletely understood, particularly at early stages of cellular differentiation. Transcriptomic approaches combined with pathway-level analyses provide an opportunity to identify coordinated biological programs that may not be evident from individual gene-level changes (Subramanian et al., 2005; Liberzon et al., 2015). Integrating these approaches with patient-specific iPSC models enables the identification of intrinsic cellular states associated with disease susceptibility.

In the present study, we generated integration-free iPSC lines from pulmonary artery smooth muscle cells obtained from a patient with sporadic PVOD who tested negative for common PVOD-associated genetic mutations. We systematically characterized these iPSC lines for pluripotency, genomic stability, and transcriptional integrity, and we assessed their capacity to differentiate into hemangioblast progenitors and endothelial cells. Rather than modeling late-stage vascular remodeling, our approach focuses on identifying early, stage-specific cellular abnormalities that may contribute to disease susceptibility. Using this framework, we identify increased oxidative stress in PVOD-derived endothelial cells emerging after progenitor differentiation, suggesting impaired endothelial homeostasis as a potential contributor to sporadic PVOD pathobiology.

## Methods

### Generation and characterization of iPSC lines

Human pulmonary artery smooth muscle cells (PASMCs) were obtained from a 60-year-old male diagnosed with pulmonary veno-occlusive disease (PVOD) based on clinical and imaging criteria. The absence of a familial form of PVOD was assessed by next-generation sequencing (NGS) analysis of genes associated with pulmonary hypertension and PVOD, including EIF2AK4, with no pathogenic variants identified. Based on this genetic evaluation and clinical presentation, the case was classified as sporadic PVOD.

Three integration-free iPSC lines were generated via nucleofection of PASMCs with episomal plasmids encoding OCT3/4, SOX2, KLF4, LIN28, and L-MYC, following the protocol described by (Okita et al., 2011). Nucleofected cells were plated onto feeder layers, and after 4 weeks, individual colonies were manually picked and expanded on Matrigel-coated plates using mTeSR1 medium. iPSC lines without genomic integration of reprogramming factors were assessed for pluripotency by qPCR for NANOG and SOX2, and by immunofluorescence for OCT4, NANOG, and SSEA-4, as previously described (Robinson et al., 2022).

Control iPSC lines (35L5, 35L11 and 37L20) were previously generated from healthy donors using the same integration-free reprogramming approach and were maintained and differentiated under identical conditions in the same laboratory to ensure comparability (Zhang et al., 2013; Freibaum et al., 2015).

### Spontaneous differentiation assay

To evaluate the pluripotent differentiation capacity, iPSC colonies were cultured on Matrigel-coated plates until reaching 60% confluency. Colonies were then dissociated into single cells and cultured in suspension to form embryoid bodies (EBs), which were maintained for 6 days. EBs were plated and allowed to spontaneously differentiate into derivatives of the three germ layers. Cells were fixed and immunostained for MAP2 (ectoderm), desmin (mesoderm), and α-fetoprotein (endoderm) as described in (Lopez-Gonzalez et al., 2019).

### Immunofluorescence

Cells were fixed in 4% paraformaldehyde for 15 min and permeabilized with 0.3% Triton X-100 for 5 min. Blocking was performed with 5% bovine serum albumin (BSA) for 1 h at room temperature. Primary antibodies were incubated overnight at 4 °C. The following primary antibodies were used (dilution 1:1000 for all): mouse anti-OCT4, rabbit anti-NANOG, mouse anti-MAP2, anti-desmin, and anti-α-fetoprotein.

### RNA extraction and quantitative PCR

Total RNA was isolated using the RNeasy Mini Kit (Qiagen), and cDNA was synthesized using the TaqMan Reverse Transcription Kit (Applied Biosystems) according to the manufacturer’s protocols. Quantitative PCR was performed using SYBR Green Master Mix (Applied Biosystems). The following primers were used: GAPDH F: TGC​ACC​ACC​ACC​TGC​TTA​GC | R: GGC​ATG​GAC​TGT​GGT​CAT​GAG; NANOG F: 5′-TTT​GTG​GGC​CTG​AAG​AAA-3' | R: 5′-AGG​GCT​GTC​CTG​AAT​AAG​CAG-3'; SOX2 F: 5′-TTC​ACA​TGT​CCC​AGC​ACT​ACC-3' | R: 5′-TCA​CAT​GTG​TGA​GAG​GGG​CAG​TGT​GC-3'. Ct values were normalized to GAPDH expression using the ΔCt method.

### Genetic analysis of iPSC lines

To assess chromosomal integrity, qPCR-based karyotype screening was performed using a kit from Stemcell Technologies. Genomic DNA (10 ng) was extracted from iPSC lines 34L2, 34L3, and 34L4 and analyzed for common chromosomal abnormalities in hESCs and iPSCs, including chromosomes 1q, 4p, 8q, 10p, 12p, 17q, 18q, 20q, and Xp, according to the manufacturer’s protocol.

### RNA sequencing and transcriptomic analysis

Total RNA was extracted from PVOD-derived iPSC lines (34L2, 34L3, 34L4) and from generated control iPSC lines (35L5, 35L11 and 37L20). RNA isolation was performed using the PureLink RNA Mini Kit (Thermo Fisher Scientific), and cDNA synthesis was carried out using the iScript cDNA Synthesis Kit (Bio-Rad). RNA-seq libraries were prepared using poly(A) selection and sequenced on an Illumina platform.

Raw sequencing data quality was assessed using FastQC (v0.12.1). High-quality reads were aligned to the GRCh38.p14 human genome using the Rsubread package (v2.18.0) in R (v4.4.1), and gene-level counts were generated for downstream analysis. Individual iPSC lines were treated as biological replicates within each group for statistical analysis.

Differential expression analysis was conducted using DESeq2 (v1.44.0). Statistical significance was assessed using the Wald test, and p-values were adjusted for multiple testing using the Benjamini–Hochberg method to control the false discovery rate (FDR). Genes with adjusted p-values (padj) < 0.05 were considered statistically significant.

Principal component analysis (PCA) was performed on normalized expression data to assess global transcriptomic relationships between samples.

Gene set enrichment analysis (GSEA) was conducted using the fgsea package in R with Hallmark gene sets obtained from the Molecular Signatures Database (MSigDB) (Subramanian et al., 2005; Liberzon et al., 2015). Genes were ranked based on the DESeq2 test statistic, and enrichment significance was evaluated using normalized enrichment scores (NES) and FDR-adjusted p-values.

Expression levels were normalized as TPM (transcripts per kilobase million) using Salmon (v1.10.1). Volcano plots were generated using ggplot2 (v3.5.1), and heatmaps were generated using ComplexHeatmap (v2.21.1). Each iPSC line represents an independent clonal derivative from the same donor and was treated as a biological replicate within the PVOD group, whereas control lines represent independent donors.

To provide orthogonal validation of transcriptomic findings, quantitative real-time PCR (qRT-PCR) was performed using independently cultured control and PVOD iPSC lines. Total RNA was isolated using the RNeasy Mini Kit (Qiagen) according to the manufacturer’s instructions. Complementary DNA (cDNA) was synthesized from 500 ng of total RNA using the High-Capacity cDNA Reverse Transcription Kit (Applied Biosystems). qRT-PCR was performed using PowerUp SYBR Green Master Mix (Applied Biosystems) on a QuantStudio 6 Flex Real-Time PCR System (Applied Biosystems). Reactions were carried out in a final volume of 10 μL containing gene-specific primers and cDNA template. Expression of representative differentially expressed genes identified by RNA sequencing (DDIT4, TXNIP, and CHCHD2) as well as oxidative stress- and TGF-β-associated genes (HMOX1 and SERPINE1) was evaluated. Gene expression was normalized to RPLP0 and analyzed using the 2^-ΔΔCt method (see Supplementary Table S3 for all primer sequences). Three independent iPSC cultures were analyzed for each cell line, and all reactions were performed in technical duplicate.

### Cell proliferation assay

iPSCs from controls and the PVOD patient were seeded onto Matrigel-coated 6-well plates. Confluency was measured at 0 and 48 h post-seeding using the Cell X robotic platform. Proliferation rate was calculated using the formula:

$$\text{Proliferation Rate}=\frac{\ln\left(\text{Confluency}\;\text{at}\;48h\;/\;\text{Confluency}\;\text{at}\;0h\right)}{48}$$

### iPSC differentiation into hemangioblast-like cells and flow cytometry analysis

Differentiation into hemangioblast-like cells followed the protocol from (Ackermann et al., 2021) with modifications. iPSC-derived embryoid bodies were cultured for 10 days in mTeSR1 medium supplemented with BMP4 (50 ng/mL), SCF (20 ng/mL), VEGF165 (50 ng/mL), and ROCK inhibitor (10 ng/mL). EBs were transferred to monolayer culture and treated with X-VIVO medium containing M-CSF (100 ng/mL) and IL-3 (25 ng/mL) to induce hematopoietic progenitor cell differentiation.

Cells were dissociated using Accutase and stained for viability with LIVE/DEAD™ Fixable Blue Dead Cell Stain (Thermo Fisher, 1:2000) for 30 min at 4 °C. Fc receptors were blocked using human TruStain FcX™ (BioLegend, 1:50), followed by antibody staining (see Supplementary Table S2) for 30 min at 4 °C. Intracellular staining was performed using a fixation/permeabilization buffer (Thermo Fisher, 00-5523-00) according to the manufacturer’s instructions.

Data were acquired using a Sony ID7000 spectral flow cytometer equipped with six lasers. Spectral unmixing was performed using UltraComp eBeads (Thermo Fisher) and autofluorescence extraction in the Sony ID7000 software. Unmixed FCS files were analyzed in FlowJo (v10.9.0).

Gating excluded debris, doublets, and dead cells, followed by sequential gating based on marker expression. A detailed gating strategy, including fluorescence minus one (FMO) controls used to define VEGFR2, CD117, CD31, and CD34 populations, is provided in Supplementary Figure S4.

Hemangioblast-like cells were defined as VEGFR2^+^/CD117^-^. Endothelial and hematopoietic lineage commitment was assessed by CD31 and CD34 expression. A minimum of 10,000 events was analyzed per sample.

### MitoSOX analysis

Mitochondrial reactive oxygen species (ROS) levels were assessed using MitoSOX™ Red (Thermo Fisher Scientific) according to the manufacturer’s instructions. Endothelial differentiation cultures were incubated with MitoSOX Red, washed, and imaged using identical acquisition settings for all samples. Nuclei were counterstained with Hoechst 33342 and used as a surrogate measure of cell number. For quantitative analysis, total MitoSOX fluorescence intensity was measured from each image and corrected for background fluorescence. Background-corrected MitoSOX signal was normalized to the number of nuclei within the corresponding field of view. Three independent differentiations were analyzed for each iPSC line.

### Western blot analysis

Western blot analysis was performed as described in (Lian et al., 2026). Briefly, endothelial differentiation cultures were lysed in Pierce™ RIPA Buffer (Thermo Scientific) supplemented with Halt™ Protease and Phosphatase Inhibitor Cocktail (Thermo Scientific). Protein concentrations were determined using the Pierce™ BCA Protein Assay Kit (Thermo Scientific), and equal amounts of protein were resolved by SDS-PAGE and transferred onto PVDF membranes. Membranes were blocked and incubated overnight at 4 °C with primary antibodies against VE-cadherin (CD144) (Cell Signaling Technology, Cat# 2158S) and β-actin (Cell Signaling Technology, Cat# 3700). Following washing with TBS-T, membranes were incubated with the appropriate IRDye® secondary antibodies (LI-COR Biosciences) for 1 h at room temperature. Protein bands were visualized using an Odyssey® DLx Imaging System (LI-COR Biosciences). Densitometric analysis was performed using Image Studio software (LI-COR Biosciences). VE-cadherin expression was normalized to β-actin and expressed relative to the mean of control endothelial cultures.

### Statistical analyses

All statistical analyses were performed using GraphPad Prism v10 (GraphPad Software, La Jolla, CA). Comparisons among multiple groups were made using one-way ANOVA followed by Tukey’s multiple comparisons test. Two-group comparisons were conducted using unpaired two-tailed Student’s t-test. Data are expressed as mean ± SEM. The number of biological replicates (n) is indicated in the corresponding figure legends. Statistical significance was defined as p < 0.05.

## Results

### Generation of iPSC lines from PASMCs

**FIGURE 1:**
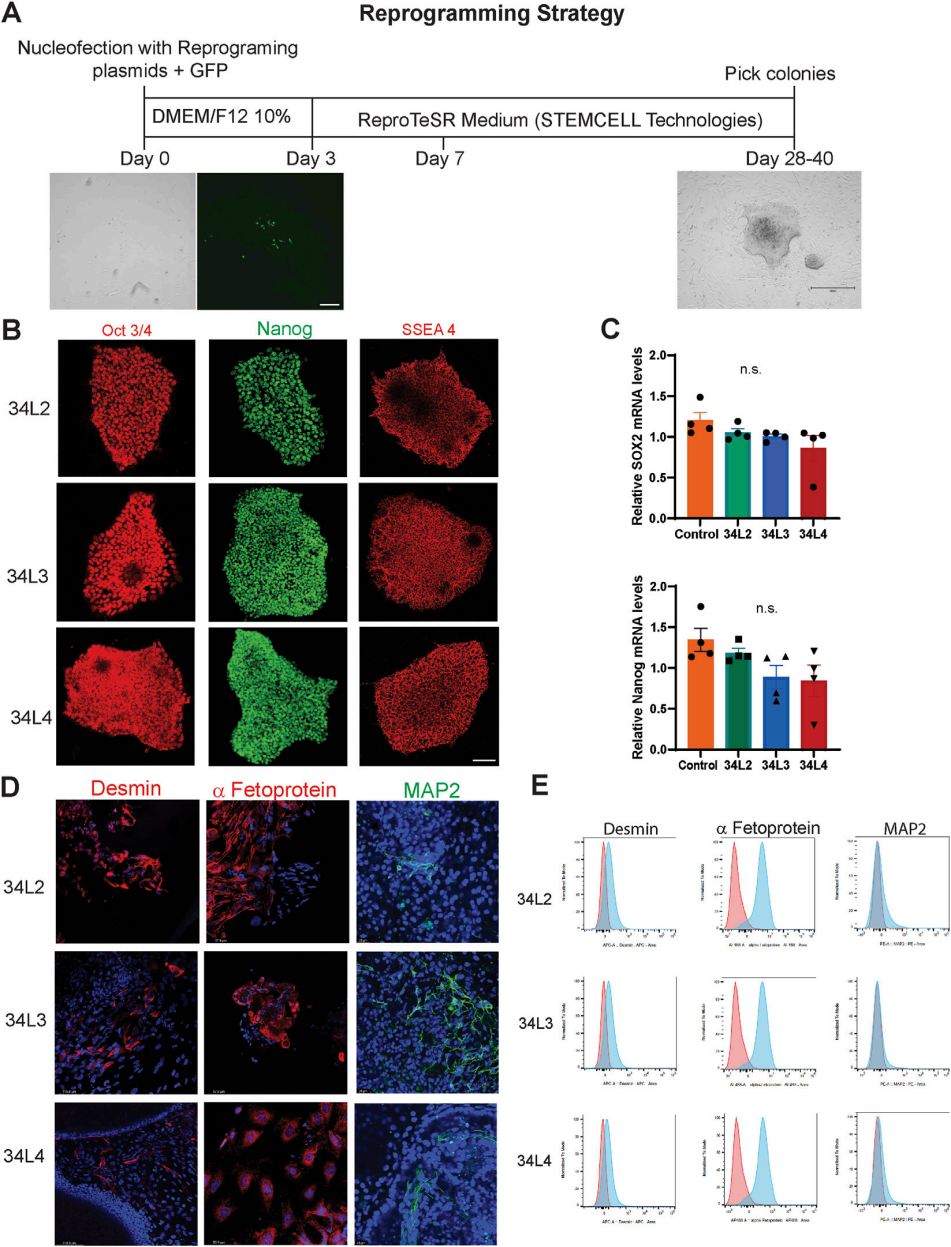
Generation and pluripotency characterization of PASMC-derived iPSC lines. **(A)** Schematic overview of the reprogramming strategy used to convert pulmonary artery smooth muscle cells (PASMCs) into induced pluripotent stem cells (iPSCs). **(B)** Representative immunofluorescence staining for pluripotency markers OCT3/4, NANOG, and SSEA-4 in newly generated PVOD-derived iPSC lines (34L2, 34L3, 34L4). Scale bar: 100 µm. **(C)** Quantitative PCR analysis of pluripotency-associated genes (SOX2 and NANOG) in PVOD-derived iPSC lines compared with a control iPSC line. Data represents four independent iPSC cultures per line and are shown as mean ± SEM. No statistically significant differences were observed (NS, non-significant). **(D)** Spontaneous differentiation of PVOD-derived iPSCs followed by immunostaining for germ layer markers: α-fetoprotein (endoderm), desmin (mesoderm), and MAP2 (ectoderm). Scale bar: 20 µm. **(E)** Flow cytometric analysis of germ layer differentiation following spontaneous differentiation. Cells were stained with lineage-specific antibodies and analyzed by flow cytometry.

Expanded PASMCs were nucleofected with a combination of episomal plasmids expressing OCT4, SOX2, KLF4, LIN28, and L-MYC using the Amaxa Nucleofection Kit number 2. To determine the optimal reprogramming conditions, we performed an optimization experiment to assess survival and nucleofection efficiency (Supplementary Figures S1A,B). Following optimization, we used the DS-137 program. Post-nucleofection, PASMCs were plated onto feeder cells in DMEM supplemented with 10% FBS. After 3 days, the medium was changed to ReproTeSR and maintained for up to 40 days. Between days 30 and 40, iPSC colonies were manually picked and transferred onto Matrigel-coated plates (Figure 1A). These colonies were expanded in 6-well plates and cryopreserved. Characterization began once iPSC lines reached passage 10. Three independent iPSC lines designated 34L2, 34L3, and 34L4 were selected for further analysis. Immunostaining for pluripotency markers Oct3/4, Nanog, and SSEA4 showed that all three iPSC lines expressed these markers (Figure 1B). Additionally, RT-qPCR analysis of SOX2 and Nanog mRNA levels showed no significant differences between the newly generated lines and a previously characterized iPSC line from a healthy control (SOX2: *p* = 0.1602; Nanog: *p* = 0.0816; Figure 1C).

### Newly generated iPSCs differentiate into derivatives of all three germ layers

To evaluate their differentiation potential, iPSC lines 34L2, 34L3, and 34L4 underwent spontaneous differentiation. Immunostaining for lineage-specific markers using desmin (mesoderm), α-fetoprotein (endoderm), and MAP2 (ectoderm) confirmed that all lines differentiated into derivatives of the three germ layers (Figure 1D). Flow cytometry analysis using the same markers also demonstrated successful differentiation into all three lineages (Figure 1E).

### PVOD iPSC lines exhibit normal karyotypes and comparable proliferation rates

**FIGURE 2:**
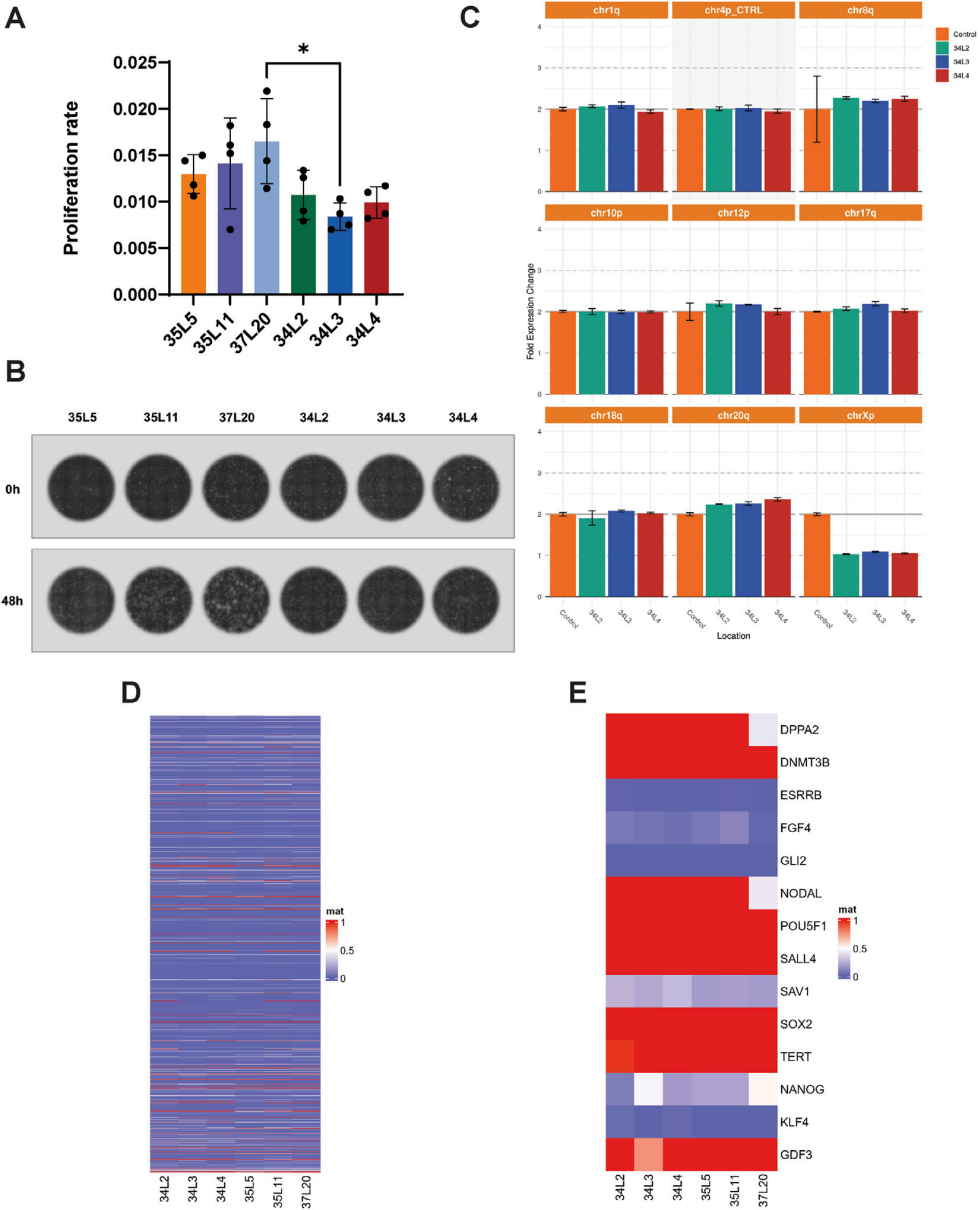
PVOD-derived iPSC lines exhibit normal proliferation, genomic stability, and preserved pluripotent transcriptional programs. **(A)** Proliferation analysis of PVOD-derived and control iPSC lines over the indicated time points. Data are presented as mean ± SEM. No significant differences in proliferation were observed between groups. However, a significant difference was observed between individual lines 37L20 and 34L3. Statistical significance was determined by one-way ANOVA. * P < 0.05. **(B)** Representative phase-contrast images showing typical iPSC colony morphology in PVOD derived and control iPSC lines. Scale bar = 20 µm. **(C)** Karyotype analysis demonstrating normal chromosomal integrity in PVOD-derived iPSC lines. **(D)** Heatmap of global gene expression profiles derived from RNA sequencing, showing overall similarity between PVOD-derived and control iPSC lines. **(E)** Heatmap of curated pluripotency-associated genes demonstrating comparable expression of key transcriptional regulators of pluripotency across PVOD-derived and control iPSC lines.

To assess proliferation, we plated iPSCs on laminin and used the CellX robotic platform to measure confluency at two time points. We analyzed PVOD lines (34L2, 34L3, 34L4) and control lines that were previously characterized (35L5, 35L11, 37L20). All newly generated lines showed similar proliferation rates. When compared to healthy controls, only 34L3 showed a statistically significant difference in proliferation relative to control line 37L20 (*p* = 0.022; Figures 2A,B; Supplementary Table S1). To evaluate karyotypic stability, we used a StemCell Technologies assay that screens for common chromosomal abnormalities in iPSCs, including regions 1q, 4p, 8q, 10p, 12p, 17q, 18q, 20q, and Xp. Genomic DNA from 34L2, 34L3, and 34L4 revealed no abnormalities, indicating normal karyotypes (Figure 2C).

### PVOD iPSCs exhibit preserved pluripotent transcriptional programs and distinct stress-associated signatures

To evaluate transcriptional features of PVOD-derived iPSCs, we performed bulk RNA sequencing and compared their expression profiles with previously characterized control iPSC lines. Global gene expression analysis demonstrated preservation of pluripotency-associated transcriptional programs across all lines, consistent with successful reprogramming and maintenance of stem cell identity. Heatmap analysis of global gene expression and curated pluripotency-associated gene sets showed comparable expression patterns between PVOD-derived and control iPSCs (Figures 2D,E; Supplementary Figure S2), in agreement with a previously described pluripotency gene network approach (Ghosh and Som, 2020).

**FIGURE 3:**
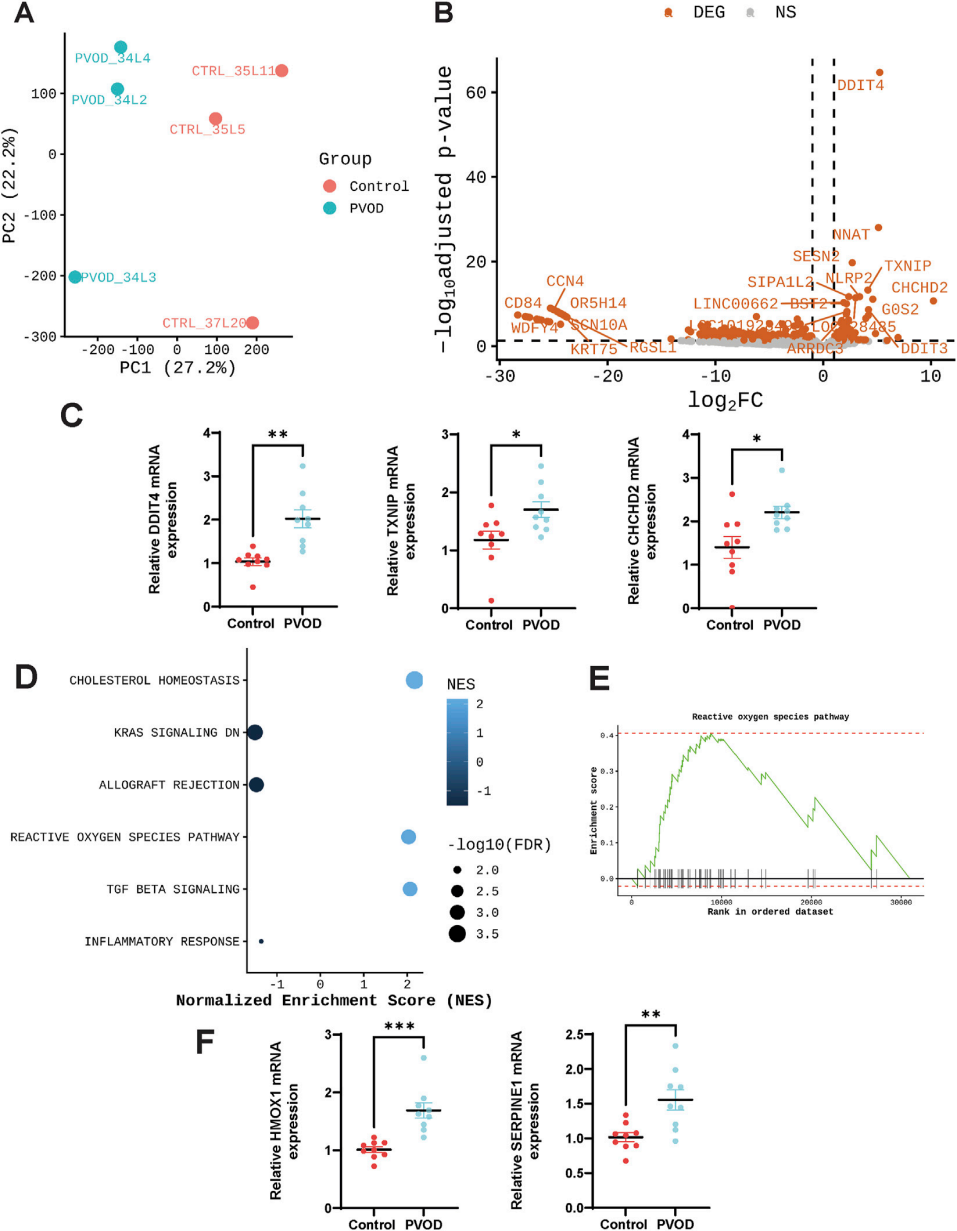
PVOD-derived iPSCs exhibit distinct differential gene expression and pathway-level transcriptional profiles. **(A)** Principal component analysis (PCA) of RNA sequencing data showing separation between PVOD-derived and control iPSC lines, indicating differences in global transcriptional profiles. **(B)** Volcano plot of differential gene expression between PVOD-derived and control iPSCs using FDR-adjusted p-values (padj). Selected differentially expressed genes are indicated. **(C)** Quantitative real-time PCR validation of representative differentially expressed genes identified by RNA sequencing DDIT4, TXNIP, and CHCHD2. Data from 3 independent iPSC cultures from 3 control and 3 PVOD lines. Bars represent mean ± SEM. Statistical significance was determined using an unpaired two-tailed Student’s t-test. * P < 0.05 and ** P < 0.01. **(D)** Gene set enrichment analysis (GSEA) of Hallmark pathways. Dot size represents −log10(FDR), and color indicates normalized enrichment score (NES). **(E)** Enrichment plot for the reactive oxygen species pathway, demonstrating pathway-level differences between PVOD-derived and control iPSCs. **(F)** Quantitative real-time PCR validation of pathway-associated genes linked to oxidative stress and TGF-β signaling (HMOX1 and SERPINE1). Data from 3 independent iPSC cultures from 3 control and 3 PVOD lines. Bars represent mean ± SEM. Statistical significance was determined using an unpaired two-tailed Student’s t-test. * P < 0.05, ** P < 0.01 and *** P < 0.001.

Despite this overall similarity, principal component analysis (PCA) revealed separation between PVOD-derived and control iPSC lines, indicating differences in global transcriptional profiles (Figure 3A). Differential expression analysis identified a subset of genes that were significantly dysregulated in PVOD-derived iPSCs relative to controls (Figure 3B). Upregulated genes included DDIT4, TXNIP, NNAT, GOS2, and CHCHD2, which are associated with cellular stress responses, redox regulation and metabolic adaptation. Conversely, several downregulated transcripts included long non-coding RNAs and lung-associated genes, such as LINC01091, TMEM51-AS1, and S100A1. To independently validate the RNA-seq findings, we performed qRT-PCR analysis of representative differentially expressed genes and pathway-associated targets using independently cultured control and PVOD iPSC lines. Consistent with the transcriptomic analysis, PVOD iPSCs exhibited significantly increased expression of DDIT4, TXNIP, and CHCHD2 compared with control iPSCs (Figure 3C).

To further investigate pathway-level alterations, we performed gene set enrichment analysis (GSEA), which identified enrichment of pathways related to oxidative stress, metabolic regulation, and signaling processes (Figure 3D). Among the positively enriched pathways in PVOD-derived iPSCs were the reactive oxygen species pathway (Figure 3E), cholesterol homeostasis, and TGF-β signaling (Supplementary Figure S3). Furthermore, qRT-PCR analysis showed that expression of HMOX1, a canonical oxidative stress-responsive gene, and SERPINE1, a downstream target associated with TGF-β signaling, were significantly elevated in PVOD iPSCs (Figure 3F). These findings provide orthogonal validation of the stress-associated transcriptional programs identified by RNA sequencing and further support the presence of altered oxidative stress and stress-response signaling in PVOD-derived iPSCs. In contrast, pathways associated with inflammatory and immune responses, including inflammatory response and allograft rejection, showed negative enrichment. Together, these findings indicate that while PVOD-derived iPSCs retain core pluripotent transcriptional features, they exhibit intrinsic transcriptional alterations involving oxidative stress, metabolic regulation, and signaling pathways.

### PVOD-derived iPSCs retain hemangioblast differentiation capacity but exhibit reduced endothelial commitment and increased oxidative stress

Given the central role of endothelial and hematopoietic lineages in PVOD pathobiology, we examined whether PVOD-derived iPSCs retained the capacity to generate hemangioblasts, early bipotent progenitors of endothelial and hematopoietic cells. Hemangioblast differentiation was induced in both control and PVOD iPSC lines, and cells were analyzed by spectral flow cytometry following exclusion of debris, doublets, and dead cells. A detailed gating strategy, including FMO controls used to define VEGFR2, CD117, CD31, and CD34 population, is provided in Supplementary Figure S4. Hemangioblast-like cells were defined as VEGFR2^+^/CD117^-^ cells according to established criteria (Teixeira et al., 2011; Rhee and Iannaccone, 2012).

**FIGURE 4:**
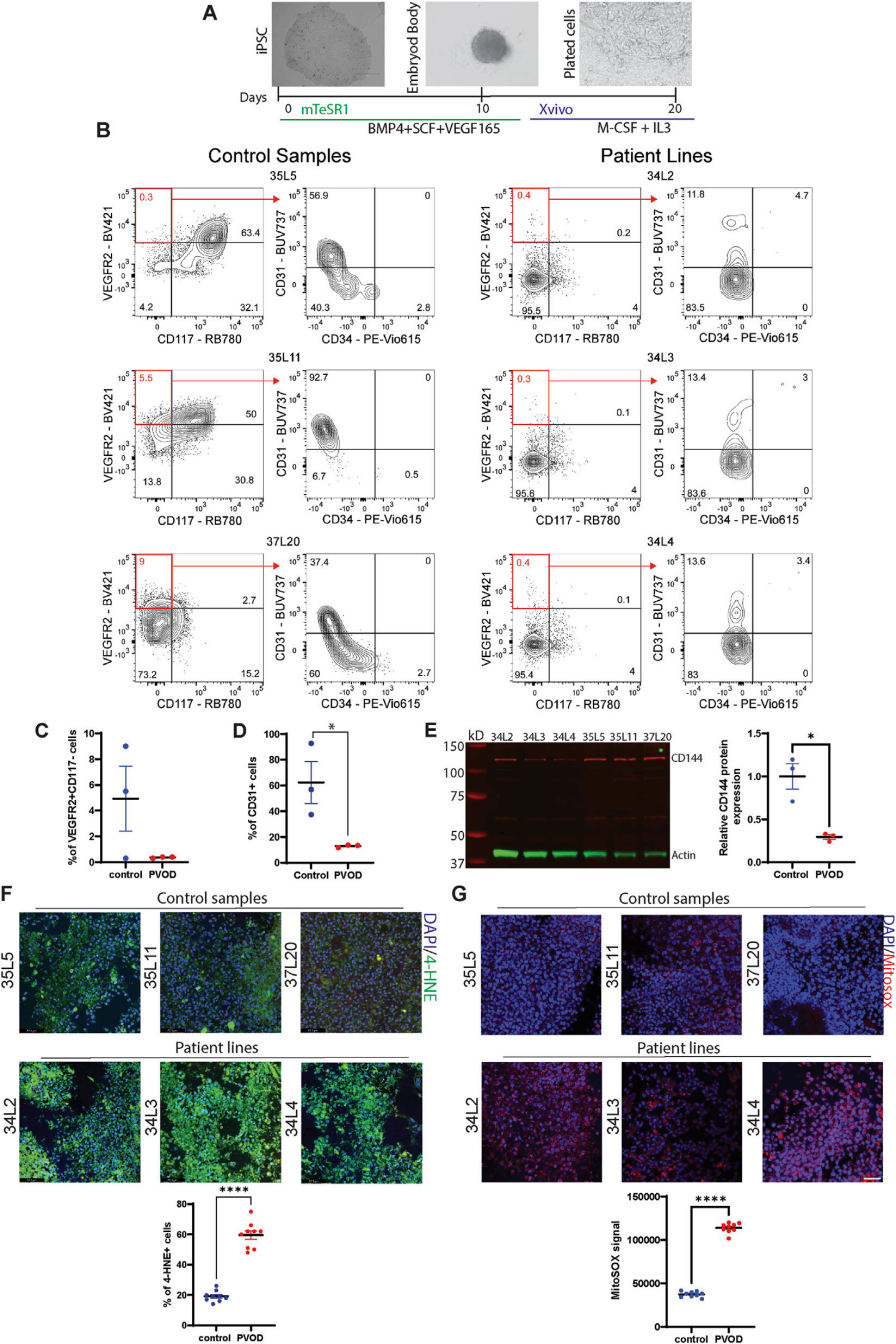
Hemangioblast differentiation capacity and oxidative stress in PVOD-derived iPSCs **(A)** Schematic overview of the differentiation strategy used to generate hemangioblast-like cells from iPSCs. **(B)** Spectral flow cytometric analysis of hemangioblast differentiation. Live, single cells were identified following exclusion of debris and doublets and downsampled to 22,816 events per sample. Hemangioblast-like cells were defined as VEGFR2^+^/CD117^-^ cells (highlighted gate). Hematopoietic/endothelial commitment was assessed by CD31 and/or CD34 expression. Quadrant values represent population frequencies. **(C)** Quantification of hemangioblast-like cell frequencies across control and PVOD-derived iPSC lines. Data are presented as mean ± SEM, with each point representing an independent iPSC line. No statistically significant differences were observed between groups. **(D)** Quantification of CD31^+^ cells as a marker of endothelial and hematopoietic lineage commitment. Data are presented as mean ± SEM, with each point representing an independent iPSC line. Statistical comparison was performed using an unpaired two-tailed Student’s t-test *P < 0.05. **(E)** Representative Western blot showing VE-cadherin (CD144) and β-actin expression in control and PVOD-derived endothelial cells. Right, densitometric quantification of VE-cadherin normalized to β-actin. Expression values were normalized to the mean of control lines. Data are presented as mean ± SEM from three control and three PVOD lines. *P < 0.05. **(F)** Representative immunofluorescence images of 4-hydroxynonenal (4-HNE) staining in endothelial cultures derived from control and PVOD iPSC lines. 4-HNE serves as a marker of lipid peroxidation and oxidative stress. Scale bar: 57.9 µm. Quantification of 4-HNE–positive cells expressed as percentage of total nuclei. Data are presented as mean ± SEM from independent cultures. ****P < 0.0001, unpaired two-tailed Student’s t-test. **(G)** Representative immunofluorescence images of MitoSOX staining in endothelial cultures derived from control and PVOD iPSC lines. Scale bar: 100 µm. Quantification of background-corrected MitoSOX fluorescence expressed as MitoSOX signal per nucleus. Data are presented as mean ± SEM from three independent differentitons per line. ****P < 0.0001, unpaired two-tailed Student’s t-test.

Control iPSC lines generated hemangioblast-like cells at variable frequencies (35L5: 0.3%; 35L11: 5.5%; 37L20: 9%), with two control lines showing notably higher values, whereas PVOD-derived lines exhibited consistently low frequencies (34L2: 0.4%; 34L3: 0.3%; 34L4: 0.4%). Although these differences did not reach statistical significance, the uniformity of low hemangioblast-like cell frequencies across PVOD-derived lines suggests that early mesodermal and hemangioblast specification is preserved but may occur within a more restricted range in PVOD iPSCs (Figures 4A–C).

To assess downstream lineage commitment, we quantified CD31 expression as a marker associated with endothelial and hematopoietic differentiation. Control lines demonstrated higher CD31 positivity (37.4%–92.7%), whereas PVOD-derived lines exhibited consistently lower CD31 expression (11.8%–13.6%). Quantitative analysis revealed a statistically significant reduction in CD31^+^ cells in PVOD-derived lines compared to controls (P < 0.05, unpaired two-tailed Student’s t-test), suggesting reduced efficiency of endothelial/hematopoietic commitment under these differentiation conditions (Figure 4D). Data are presented as mean ± SEM, with each point representing an independent iPSC line, and should be interpreted cautiously given the limited number of lines and the use of clonal replicates for the PVOD group. To further evaluate endothelial lineage-associated marker expression, we assessed VE-cadherin (CD144) protein levels in differentiated endothelial cultures by Western blot analysis. PVOD-derived endothelial cultures exhibited reduced CD144 expression compared with control cultures. Densitometric quantification normalized to β-actin demonstrated an approximately 70% reduction in CD144 protein levels in PVOD-derived cells (Figure 4E). Together with the observed reduction in CD31-positive cells, these findings support altered endothelial marker expression in PVOD-derived endothelial cultures.

Because transcriptomic analyses identified enrichment of oxidative stress–related pathways in PVOD iPSCs, we next assessed oxidative stress at the protein level. Immunostaining for 4-hydroxynonenal (4-HNE), a marker of lipid peroxidation and oxidative damage, revealed increased 4-HNE immunoreactivity in PVOD-derived cultures compared to controls under endothelial media conditions, consistent with elevated lipid peroxidation in these cultures (Figure 4F). To further assess oxidative stress, mitochondrial ROS levels were measured using MitoSOX. Consistent with the increased 4-HNE staining, PVOD-derived endothelial cells exhibited significantly increased MitoSOX fluorescence compared with controls, demonstrating an approximately 3-fold increase in mitochondrial ROS levels (Figure 4G). These findings provide additional evidence of altered redox homeostasis in PVOD-derived cells and are consistent with activation of oxidative stress pathways identified by RNA sequencing.

## Discussion

Pulmonary veno-occlusive disease (PVOD) is a rare and severe form of pulmonary hypertension characterized by progressive obstruction of small pulmonary veins and venules, ultimately leading to increased pulmonary vascular resistance and right heart failure (Montani et al., 2016; Humbert et al., 2022). Despite advances in understanding the genetic basis of heritable PVOD, including the identification of EIF2AK4 mutations (Eyries et al., 2014), the mechanisms underlying sporadic cases remain poorly defined. In this study, we generated induced pluripotent stem cells (iPSCs) from a patient with sporadic PVOD and used this model to investigate early cellular and molecular features associated with the disease.

Our results demonstrate that PVOD-derived iPSCs retain key characteristics of pluripotency, including typical colony morphology, expression of pluripotency markers, and the capacity to differentiate into derivatives of the three germ layers. In addition, proliferation rates and karyotype analyses indicated no overt abnormalities, supporting the stability of the reprogrammed lines. Transcriptomic analyses further confirmed that PVOD-derived iPSCs maintain global and pluripotency-associated gene expression programs comparable to control iPSCs, consistent with previous studies describing robust maintenance of pluripotency networks following reprogramming (Buganim et al., 2012; Ghosh and Som, 2020). These findings reinforce the suitability of these cells as a model system to investigate disease-relevant mechanisms.

Despite the preservation of pluripotent identity, transcriptomic profiling revealed distinct differences between PVOD-derived and control iPSCs. Principal component analysis demonstrated separation between the two groups, indicating global transcriptional differences. Differential expression analysis identified genes associated with cellular stress, redox regulation, and metabolic adaptation, including DDIT4, TXNIP, NNAT, GOS2, and CHCHD2, as being upregulated in PVOD-derived iPSCs. These findings suggest that disease-associated molecular alterations are present even in the undifferentiated state, consistent with prior reports showing that patient-derived iPSCs can retain disease-relevant transcriptional signatures (Grandy et al., 2019; Gu et al., 2017).

At the pathway level, gene set enrichment analysis revealed enrichment of pathways related to oxidative stress, metabolic regulation, and signaling processes, including the reactive oxygen species pathway, cholesterol homeostasis, and TGF-β signaling, alongside relative downregulation of inflammatory and immune-related pathways. Importantly, independent qRT-PCR validation confirmed the upregulation of representative stress-associated genes identified by RNA sequencing, providing orthogonal support for the oxidative stress-related transcriptional alterations observed in PVOD-derived iPSCs.

The enrichment of oxidative stress-related pathways is further supported by increased levels of 4-hydroxynonenal (4-HNE), a marker of lipid peroxidation, and elevated mitochondrial ROS levels measured by MitoSOX in PVOD-derived cells. Oxidative stress has been extensively implicated in pulmonary vascular disease and vascular remodeling (Bowers et al., 2004; Zelko et al., 2018), supporting the biological relevance of these observations. Collectively, these findings are consistent with altered redox homeostasis in PVOD-derived cells.

The identification of hemangioblast-like populations in this study was based on the expression of VEGFR2 (KDR), a well-established marker of early mesodermal and hemangiogenic progenitors. Hemangioblasts are defined as common precursors of endothelial and hematopoietic lineages and have been shown to express shared markers, including VEGFR2, GATA factors, and TAL1, particularly within mesodermal populations contributing to blood island formation (Teixeira et al., 2011). However, a strict consensus definition of hemangioblasts based on a single marker combination remains lacking, and marker expression is known to be dynamic and context-dependent during early development (Rhee and Iannaccone, 2012). In this context, CD117 (c-KIT) expression has been reported to vary across developmental stages and progenitor subsets. Therefore, we defined VEGFR2^+^/CD117^-^ cells as a hemangioblast-like population, reflecting an early endothelial-primed progenitor state rather than a canonical or fully defined hemangioblast population.

The observed enrichment of TGF-β signaling pathways is also notable, as this pathway has been implicated in vascular remodeling and endothelial dysfunction in pulmonary hypertension (Rabinovitch et al., 2014). Similarly, alterations in cholesterol homeostasis pathways may reflect broader metabolic adaptations that contribute to disease susceptibility or progression. While these findings do not establish causality, they suggest that PVOD-derived iPSCs harbor intrinsic transcriptional features that may predispose cells to pathological responses upon differentiation.

Importantly, the use of undifferentiated iPSCs in this study allows for the identification of early, cell-intrinsic transcriptional alterations that are independent of lineage-specific effects. However, this approach also represents a limitation, as the functional consequences of these transcriptional changes in disease-relevant cell types, such as endothelial cells, remain to be fully characterized. Future studies involving directed differentiation and functional assays will be necessary to determine how these molecular signatures influence vascular cell behavior and contribute to PVOD pathogenesis. Recent work using patient-specific iPSC-derived endothelial cells further supports the utility of this approach for modeling pulmonary vascular disease (Ma et al., 2022).

In summary, our study demonstrates that iPSCs derived from a patient with sporadic PVOD retain pluripotent identity while exhibiting distinct transcriptional alterations involving oxidative stress, metabolic pathways, and signaling processes. These findings support the use of patient-derived iPSCs as a platform to investigate early disease-associated mechanisms and provide a foundation for future studies aimed at elucidating the cellular basis of PVOD.

## Data Availability

The datasets presented in this study can be found in online repositories. The names of the repository/repositories and accession number(s) can be found in the article/Supplementary Material.
