# Supplementary material for: Induced pluripotent stem cells from sporadic pulmonary veno-occlusive disease exhibit altered endothelial commitment and stress-related transcriptional programs
Source: Front Cell Dev Biol. 2026 Aug 3;14:1811995. doi: 10.3389/fcell.2026.1811995 (PMC13478140; doi:10.3389/fcell.2026.1811995)
Supplement: Supplementary file 1 [file Supplementaryfile1.pdf]

Supplementary Tables and Figures

Supplementary Table 1. Automated confluency measurements of iPSCs from 3 healthy controls and 3 PVOD lines using the cellX system.

**Line number**	35L5		35L11		37L20		34L2		34L3		34L4	
**Timepoint**	0h	48h	0h	48h	0h	48h	0h	48h	0h	48h	0h	48h
**Confluency**	17%	30%	24%	52%	22%	63%	24%	37%	35%	49%	31%	46%

Supplementary Table 2. List of antibodies used for flow cytometry experiments.

**Antibody**	**Fluorochrome**	**Location**	**Vendor**	**Catalog #**	**Clone**	**Concentration**	**Final Dilution**
VEGFR2	BV421	Cell Surface	BioLegend	393009	A16085H	100 µg/mL	1/160
CD117	RB780	Cell Surface	Becton Dickinson	569064	104D2	100 µg/mL	1/80
CD31	BUV737	Cell Surface	Becton Dickinson	741834	WM59	200 µg/mL	1/80
CD34	PE-Vio 615	Cell Surface	Miltenyi Biotec	130-120-521	REA1164	100 µg/mL	1/400
Desmin	APC	Cell Surface	Miltenyi Biotec	130-119-491	REA1134	75 µg/mL	1/200
MAP2	PE	Cell Surface	Miltenyi Biotec	130-128-142	REAL1048	250 µg/mL	1/40
Alpha-fetoprotein	AF488	Intracellular	R&D Systems	IC1369G	189506	200 µg/mL	1/100

Supplementary Table 3. Primer sequences used for quantitative real-time PCR validation.

**Gene**	**Forward Primer (5'→3')**	**Reverse Primer (5'→3')**
DDIT4	GTTTGACCGCTCCACGAGCCT	GCACACAAGTGTTCATCCTCAGG
TXNIP	CAGCAGTGCAAACAGACTTCGG	CTGAGGAAGCTCAAAGCCGAAC
CHCHD2	GTGGAGGAAGTAATGCTGAGCC	CACAGAGCTTGATGTCACCCTG
HMOX1	CCAGGCAGAGAATGCTGAGTTC	AAGACTGGGCTCTCCTTGTTGC
SERPINE1	CTCATCAGCCACTGGAAAGGCA	GACTCGTGAAGTCAGCCTGAAAC
RPLP0	TGGTCATCCAGCAGGTGTTCGA	ACAGACACTGGCAACATTGCGG

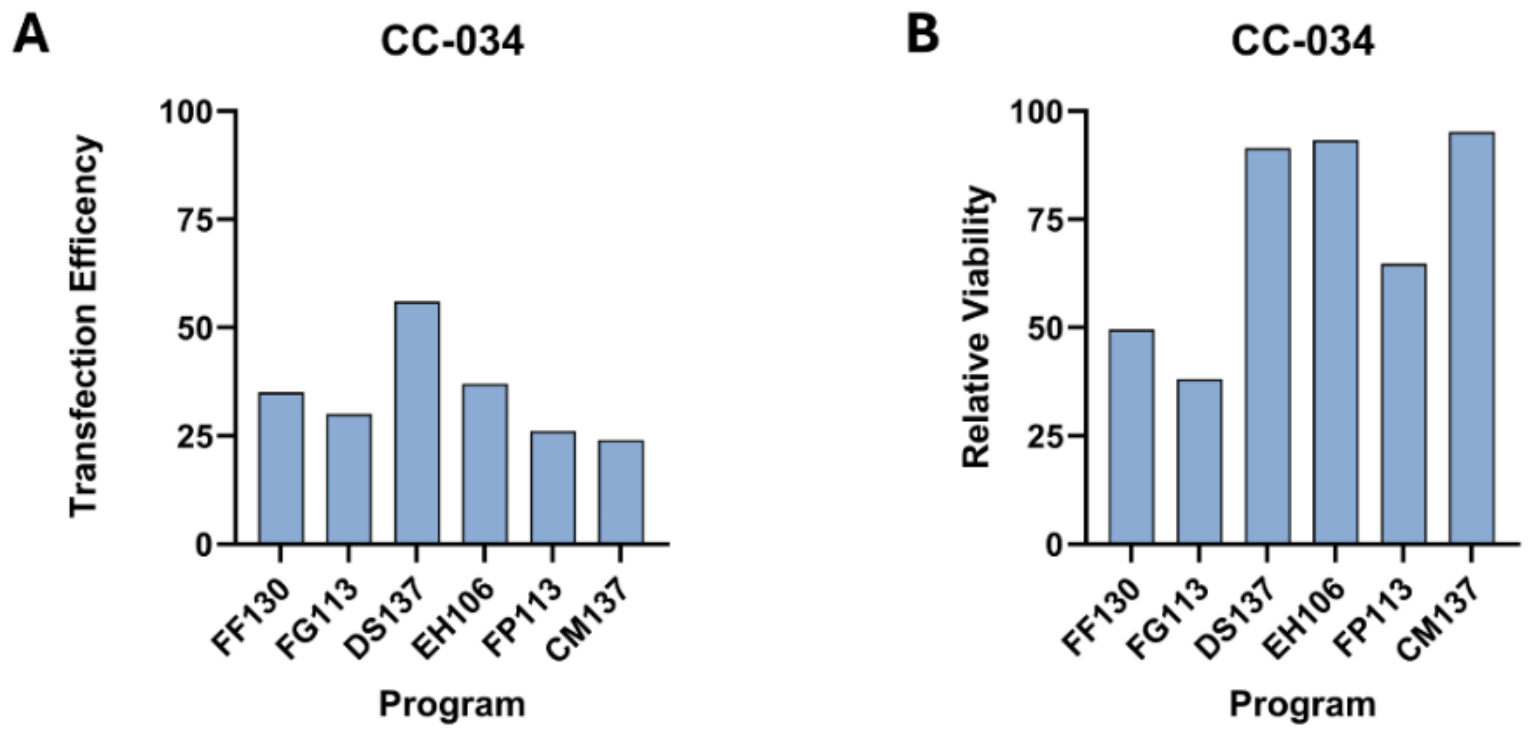

Supplementary Figure 1. Nucleofection optimization of PASMCs. We nucleofected PASMCs using the Amaxa nucleofection kit number 2 and analyzed nucleofection efficiency (1 A) and cell relative survival (1 B) using different programs.

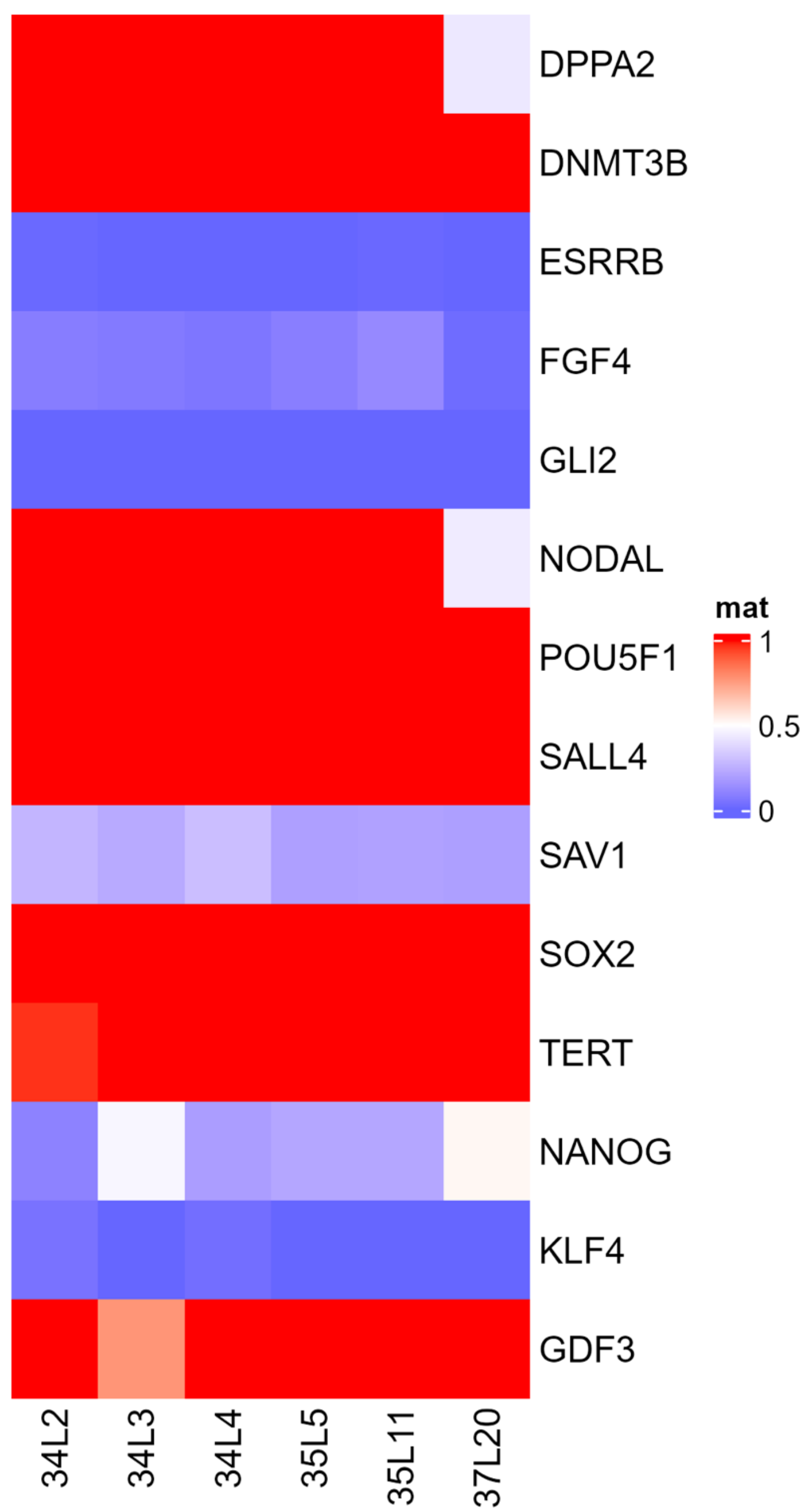

Supplementary Figure 2. Heatmap of pluripotent genes expressed in iPSCs from controls and PVOD lines. Heatmap of gene expression patterns of pluripotent markers from control and patient lines. Color bars demonstrate transcripts per million (TPM) from lowest (purple) to highest (red) for each gene across each line, showing that the pluripotent gene expression tendency between controls and patients are consistent, while there are slight differences in gene expression between different lines from the same individual (34L2, 34L3, 34L4).

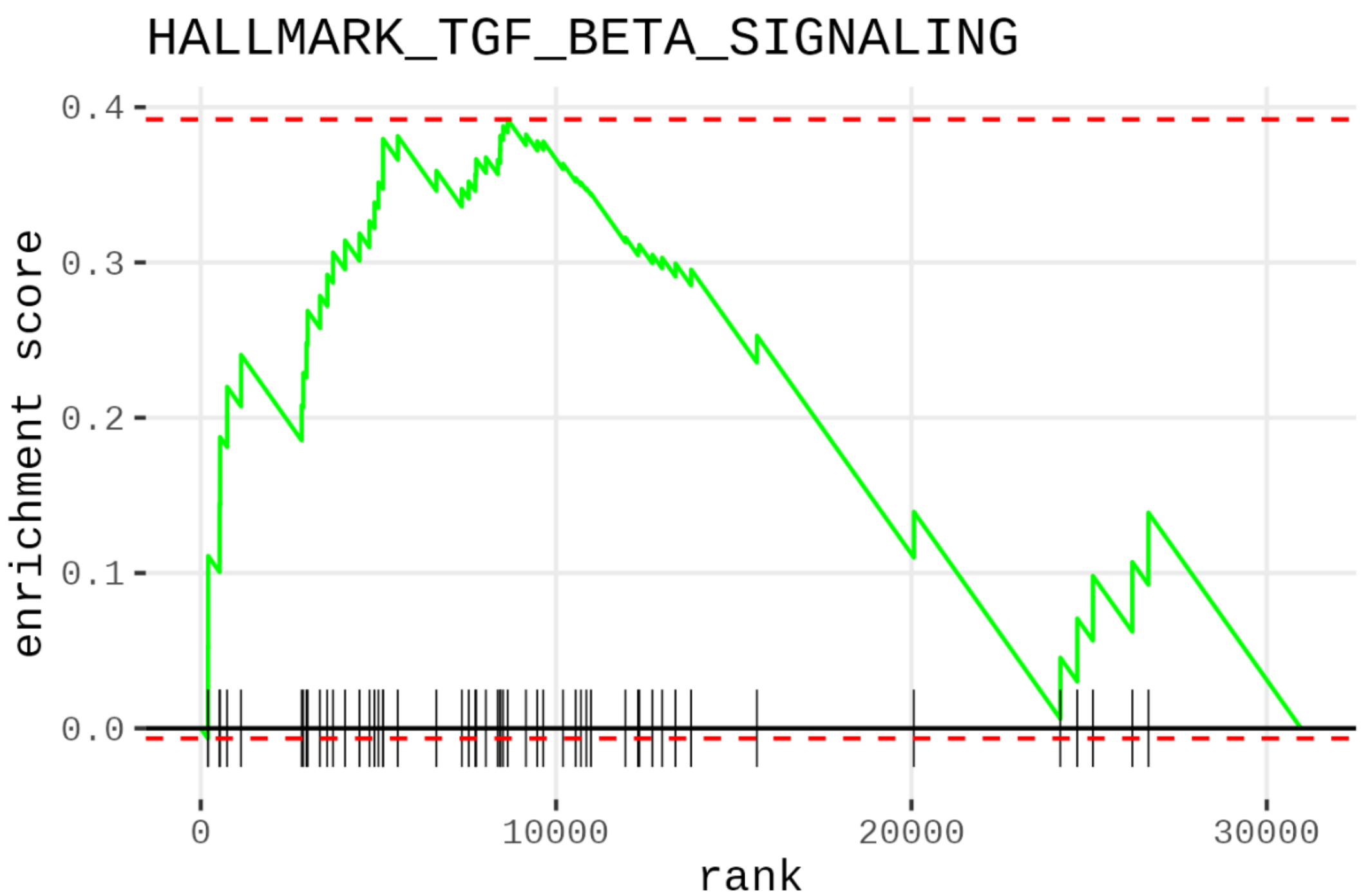

Supplementary Figure 3. Enrichment of TGF-β signaling pathway in PVOD-derived iPSCs. Gene set enrichment analysis (GSEA) enrichment plot for the Hallmark TGF-β signaling pathway based on preranked differential expression analysis. The enrichment score (ES) is plotted across the ranked gene list, with genes contributing to the enrichment signal indicated along the curve. The distribution of pathway-associated genes within the ranked dataset is shown below the enrichment curve.

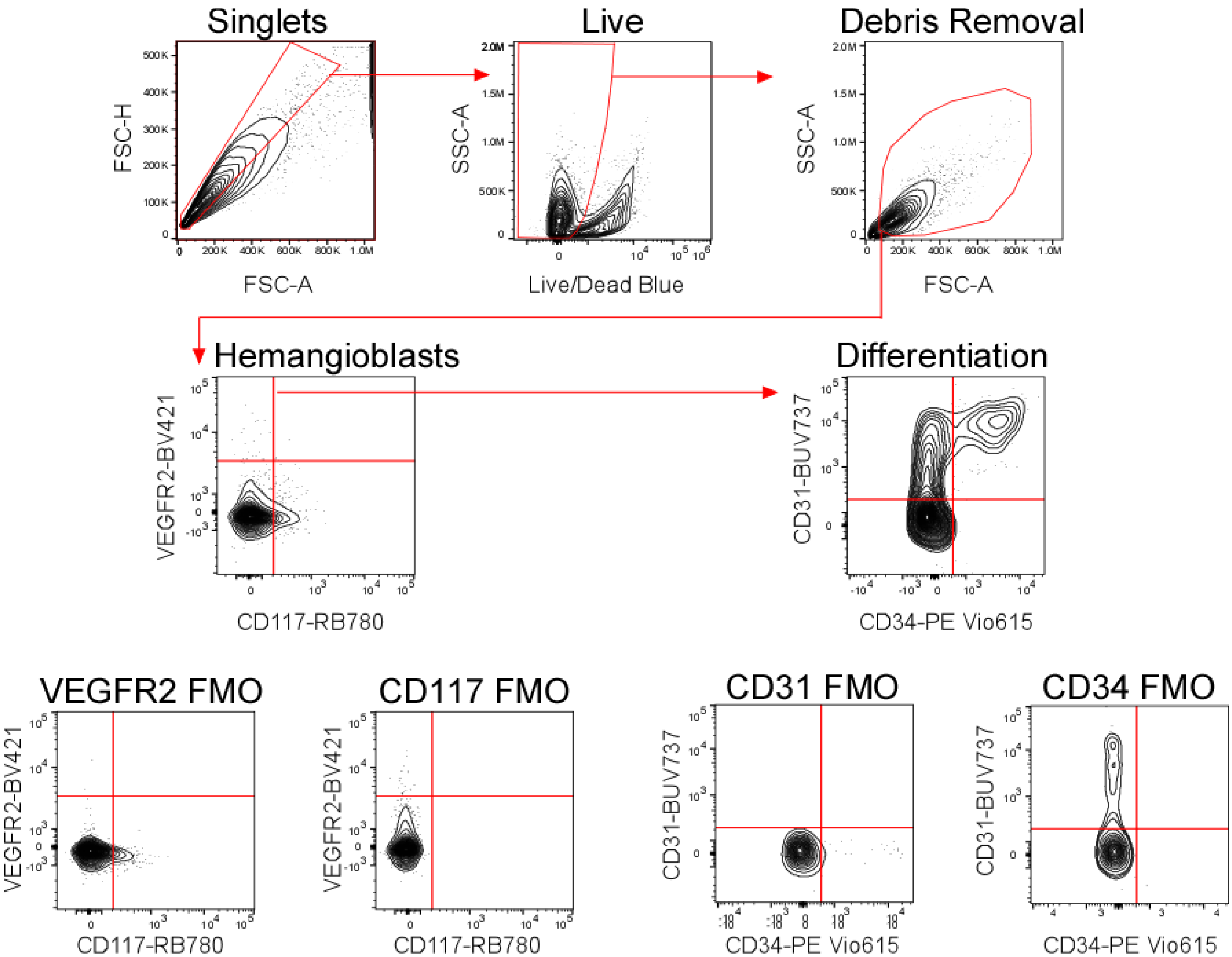

Supplementary Figure 4. Flow cytometry gating strategy including fluorescence minus one (FMO) controls. Data clean-up included exclusion of doublets, dead cells, and debris. VEGFR2+/CD117- hemangioblasts were identified, and then hematopoietic commitment of the hemangioblasts was determined by the expression of CD31 and/or CD34. Fluorescence minus one (FMO) samples for VEGFR2 and CD117 were used to gate hemangioblasts, and FMOs for CD31 and CD34 were used to gate for hematopoietic commitment.
